# Dietary *Fagopyrum dibotrys* Extract Supplementation: Impacts on Growth Performance, Immune Response, Intestinal Morphology, and Microbial Community in Broiler Chickens Infected with *Escherichia coli* O157

**DOI:** 10.3390/ani15243515

**Published:** 2025-12-05

**Authors:** Jiang Chen, Gaoxiang Ai, Pingwen Xiong, Wenjing Song, Guohua Liu, Qipeng Wei, Xiaolian Chen, Zhiheng Zou, Qiongli Song

**Affiliations:** 1 Jiangxi Province Key Laboratory of Animal Green and Healthy Breeding, Institute of Animal Husbandry and Veterinary Science, Jiangxi Academy of Agricultural Sciences, Nanchang 330200, China; jiangchen@jxaas.cn (J.C.); aigaoxiang@jxaas.cn (G.A.); xiongpingwen1990@jxaas.cn (P.X.); wenjingsong@jxaas.cn (W.S.); weiqp66@jxaas.cn (Q.W.); chenxl@jxaas.cn (X.C.); zouzh@jxaas.cn (Z.Z.); 2 Key Laboratory of Feed Biotechnology of the Ministry of Agriculture, Institute of Feed Research, Chinese Academy of Agricultural Sciences, Beijing 100081, China; liuguohua@caas.cn

**Keywords:** broilers, *Fagopyrum dibotrys* extract, *Escherichia coli*, growth performance, intestinal inflammation

## Abstract

This study explored the efficacy of dietary *Fagopyrum dibotrys* extract (FDE) in mitigating *Escherichia coli* O157 (*E. coli*) infections in broilers. A total of 240 one-day-old male Shengze 901 broilers were randomly allocated to four groups (with 10 broilers per group): CON (basal diet), COLI (basal diet + *E. coli* challenge), FDE (basal diet + 500 mg/kg FDE), and FDEC (basal diet + 500 mg/kg FDE + *E. coli* challenge). The results showed that *E. coli* challenge reduced the average daily gain (ADG) and average daily feed intake (ADFI), increased the feed conversion ratio (FCR) and cecal *E. coli* load, impaired the intestinal mucosa, and induced intestinal inflammatory responses (*p* < 0.05). FDE supplementation improved growth performance, increased duodenal villus height and villus/crypt ratio; reduced serum interleukin (IL)-1β, tumor necrosis factor-α (TNF-α), diamine oxidase (DAO), and endotoxin levels; and lowered cecal *E. coli* counts (*p* < 0.05). Molecularly, FDE supplementation upregulated Occludin, Claudin-1, and ZO-1 gene expressions, and downregulated jejunal TLR4 and MyD88 mRNA levels. Microbiome analysis revealed that FDE increased the relative abundance of *Faecalibacterium* and alleviated the *E. coli*-induced reduction in *Clostridia_UCG-014*. In conclusion, dietary supplementation with 500 mg/kg FDE could mitigate colibacillosis-related intestinal damage and inflammatory responses.

## Simple Summary

Avian pathogenic *Escherichia coli* is the most prevalent and damaging bacterial disease in chickens, causing significant economic losses in the poultry industry. With the implementation of measures banning the use of feed antibiotics in the poultry industry worldwide, there has been an urgent need to develop new technologies and products to replace antibiotics for the prevention and control of avian *Escherichia coli* disease. Therefore, this study investigated the effects of dietary *Fagopyrum dibotrys* extract supplementation on alleviating *Escherichia coli* infection by constructing an in vivo model of *Escherichia coli* infection in broiler chickens. We found that adding 500 mg/kg of *Fagopyrum dibotrys* extract to the feed successfully alleviated the intestinal damage and inflammation caused by *Escherichia coli* infection in chickens.

## 1. Introduction

Avian pathogenic *Escherichia coli* (APEC) infection has an incidence rate of 9–36% in commercial broiler flocks worldwide, with a mortality rate of 5–10% in infected flocks [1,2]. APEC has been causing economic losses exceeding hundreds of millions of US dollars annually for decades, primarily due to reduced production performance, increased treatment costs, and the disposal of carcasses [3,4]. Following the implementation of China’s antibiotic growth promoter (AGP) prohibition in animal feed effective July 2020 [5], gastrointestinal health management has emerged as a critical challenge in poultry production. Consequently, the poultry industry faces increasing pressure to develop eco-friendly, effective, and safe antibiotic alternatives for bacterial infection prevention and immunity enhancement. Numerous herbal products and their extracts are one of the main alternative strategies used as growth-promoting feed additives in livestock production [6,7,8]. Herbal plant extracts contain rich amounts of alkaloids with anti-disease properties, flavonoids with antioxidant properties, and phenolic compounds [9,10]. Their active components can promote animal growth by regulating immune function, enhancing antioxidant properties, and reducing inflammatory responses.

*Fagopyrum dibotrys*, a perennial herbaceous plant, is primarily cultivated in Asia. To date, researchers have successfully extracted and characterized over 100 distinct chemical substances from this botanical species, primarily categorized as flavonoids, phenolic substances, terpenoids, steroids, and fatty acids [11]. Research indicates that flavonoids and phenolic substances represent the principal bioactive components within this plant [11,12,13]. Previous pharmacological studies have revealed that *Fagopyrum dibotrys* displays anti-inflammatory, antioxidant, and antidiabetic activities [14,15,16]. *Fagopyrum dibotrys* has also been shown to possess notable antibacterial properties effective against multiple bacterial strains, such as *E. coli* and *Salmonella* species [11]. *Fagopyrum dibotrys* is officially recognized as a permitted feed component in China’s Feed Ingredient Catalog. As a feed ingredient, *Fagopyrum dibotrys* has advantages such as high utilization value and no drug resistance [17]. However, its application in feed is limited by complex processing procedures and unclear optimal dosage for different livestock species. Investigating the effects of *Fagopyrum dibotrys* extract (FDE) on livestock and poultry health is a feasible approach, as extraction can simplify its application in feed and facilitate the determination of optimal dosage.

FDE has been shown to alleviate human intestinal inflammation and enhance intestinal mucosal epithelial function by increasing the expression of tight junction proteins Claudin-1, Claudin, and ZO-1 in the colonic epithelial cells of patients with irritable bowel syndrome [18]. FDE demonstrated the ability to improve antioxidant function in the intestines by activating the nuclear factor erythroid 2-related factor 2 (NRF2) signaling pathway in an oxidatively stressed chicken model [19]. Currently, the efficacy and mechanism of FDE in alleviating *E*. *coli* infection in broiler chickens remain unclear. Therefore, this study aims to evaluate the effects of FDE on growth performance, intestinal inflammatory responses, barrier integrity, and microbial composition in *E. coli* O157-challenged broilers. The findings will provide valuable insights and a theoretical basis for utilizing FDE to mitigate intestinal inflammation caused by *E. coli* infection.

## 2. Materials and Methods

The FDE was purchased from Lanzhou Wotelaisi Biological Technology Co., Ltd. (Lanzhou, China), with a flavonoid content of 9.34 mg/g and a total phenolic content of 4.21 mg/g.

### 2.1. Experimental Design, Diets, and Animal Management

A 2 × 2 randomized factorial experimental design was implemented to assess the impact of FDE supplementation (0 or 500 mg/kg of diet) and *E. coli* challenge (challenged or non-challenged). A total of 240 one-day-old male Shengze 901 broilers were randomly allocated to 4 groups, with 6 replicates per group and 10 broilers per replicate (experimental unit: replicate cage). The four groups were as follows: (1) CON group: basal diet without *E. coli* challenge; (2) COLI group: basal diet + *E. coli* O157 challenge; (3) FDE group: basal diet supplemented with 500 mg/kg FDE without *E. coli* challenge; (4) FDEC group: basal diet supplemented with 500 mg/kg FDE + *E. coli* O157 challenge. The test was carried out for 21 days. The basal diet was a corn-soybean meal-based diet, formulated in accordance to the chicken feeding standard NY/T33-2004 [20]. The basal diet nutritional level is shown in Table 1.

This experiment was carried out at the Farm of the Institute of Animal Science and Veterinary Medicine, Jiangxi Academy of Agricultural Sciences. Before starting the experiment, the entire chicken house and experimental equipment were thoroughly cleaned and disinfected. Broilers were reared in three-tier cages (70 cm length × 70 cm width × 50 cm high) with 5 broilers per cage. Each replicate (10 broilers) was housed in 2 adjacent cages to ensure uniform environmental conditions. Each pen contained single-tube feeders and 2 nipple drinkers providing the chickens with ad libitum access to feed and water. The environmental control parameters were set based on previous studies [21,22]. Environmental controls maintained an initial temperature regime of 33 °C during the first 72 h, which was gradually reduced by 2 °C each subsequent week. The chicken house was equipped with a forced ventilation system, and relative humidity was maintained at 60–65% throughout the experiment. Lighting protocols consisted of constant illumination for the initial 72 h, transitioning to an 18 h photoperiod with 6 h of darkness from day 4 through day 21, maintained at 5 lux intensity. During the rearing process, detailed records were kept, and any abnormalities were addressed promptly.

### 2.2. Escherichia coli O157 Challenge

For the infection model, the bacterial strain used for infection was *E. coli* O157, purchased from the China Animal Health and Veterinary Development Center (CAU0771). The bacterial culture was prepared by introducing *E. coli* O157 into LB broth (Qingdao Hope Bio-Technology Co., Ltd., Qingdao, China) followed by incubation at 37 °C with continuous agitation (180 rpm) over 24 h. Bacterial growth monitoring involved collecting 1 mL samples at two-hour intervals for optical density measurement, with parallel cell counting performed using a hemocytometer chamber to determine colony concentrations. From experimental days 9 to 11, the broilers in COLI and FDEC groups were orally administered 0.5 mL of *E. coli* O157 solution (3 × 10^8^ colony-forming unit (CFU)/mL) via pipette, according to the method described [23]. Concurrently, the CON and FDE groups were administered equivalent volumes of sterilized LB broth as a placebo treatment.

### 2.3. Growth Performance

After 12 h of fasting, the body weight (BW) of broilers was measured per replicate during morning hours on day 14 and day 21. Feed consumption data were documented per replicate throughout the experimental period. Subsequently, the average daily gain (ADG), average daily feed intake (ADFI), and feed conversion ratio (FCR) were calculated.

### 2.4. Sample Collection

At 3 days post inoculation (DPI) of *E. coli* (d 14), one broiler with a body weight close to the average weight of the replicate was selected. Approximately 4 mL blood was collected from the wing vein into 5 mL VACUETTE tubes (Hebei Kangweishi Medical Technology Co., Ltd., Shijiazhuang, China), and subjected to centrifugation (1500× *g*, 10 min, 4 °C). Then, the serum samples were aliquoted and maintained at −20 °C until analysis. At 21 days of age, one bird was selected from each replicate and euthanized by head-only electrical stunning, and then exsanguinated via two-sided neck cut. Mid-duodenal and mid-jejunal segments (1.5 cm each) were excised, gently flushed with sterile physiological saline (0.9% NaCl, pH 7.4, Beijing Dingguo Changsheng Biotechnology Co., Ltd., Beijing, China), and fixed in a 4% formaldehyde solution (prepared with phosphate-buffered saline, Wuhan Servare Biotechnology Co., Ltd., Wuhan, China) for histological preservation [24]. The formalin-fixed intestinal segments were processed into 5 μm histological sections, subsequently stained with hematoxylin and eosin (H&E) reagents (Shanghai Yuanye Biotechnology Co., Ltd., Shanghai China), and analyzed using light microscopy (BA210Digital, Motic Microscopes, Beijing, China) at 400× magnification. Ten random fields with intact villi and crypts and good orientation were selected from each slice, and the villus height (VH) and crypt depth (CD) were measured (Motic Images Advanced 3.2). The VH/CD ratios were calculated using Microsoft Excel 2019. Concurrently, segments of jejunal tissue (3 cm each) were rapidly excised, rinsed with sterile physiological saline, blotted dry with filter paper, and immediately stored in liquid nitrogen for subsequent RNA extraction. Approximately 1 g of contents from the cecum was collected and gently squeezed into sterile tubes for microbiological analysis. Subsequently, all samples were flash frozen in liquid nitrogen and stored at −80 °C until being used for DNA collection.

### 2.5. ELISA for the Measurement of Cytokines

Serum concentrations of interleukin (IL)-1β, IL-10, tumor necrosis factor-α (TNF-α), serum diamine oxidase (DAO) activity, and serum endotoxin (LPS) were measured using commercial chicken-specific ELISA kits (Shanghai Enzyme-linked Biotechnology Co., Ltd., Shanghai, China), following the manufacturer’s instructions.

### 2.6. Real-Time Quantitative PCR Analysis

Total RNA extraction from ileum tissues was performed with the Trizol reagent (Invitrogen, Carlsbad, CA, USA). RNA purity and yield were evaluated with a NanoDrop-2000 spectrophotometer (ThermoFisher Scientific, Waltham, MA, USA) through OD260/280 ratio measurements (target range: 1.8–2.0). RNA integrity was verified using 1.5% denaturing agarose gel electrophoresis run at 120 V for 30 min, with clear 28S and 18S rRNA bands indicating intact RNA. Reverse transcription of RNA into complementary DNA was carried out using the TransScript First-Strand cDNA Synthesis Kit (TransGen Biotech, Beijing, China), which uses oligo(dT) primers. Two negative controls were included to validate cDNA quality: (1) RT-control: reaction system without reverse transcriptase, to detect genomic DNA contamination, and (2) no-template control (NTC): reaction system without RNA template, to exclude reagent or environmental contamination. All controls were run in parallel with samples, and no amplification was observed in either control, confirming no contamination and reliable cDNA synthesis. Specific primer sequences were designed through analysis in the NCBI GenBank database and commercially produced by Sangon Biotech (Shanghai, China), with detailed primer information and gene accession numbers provided in Table 2.

Real-time qPCR was performed in a 20 μL reaction volume containing 10 μL MagicSYBR Mixture (Beijing Cowin Biotech, Beijing, China), 0.4 μL forward primer (10 μmol/L), 0.4 μL reverse primer (10 μmol/L), 2 μL cDNA template (100 ng/μL), and 7.2 μL nuclease-free water. The amplification program was as follows: initial denaturation at 95 °C for 5 min, 40 cycles of denaturation at 95 °C for 10 s, annealing at 60 °C for 30 s, and extension at 72 °C for 5 min; then, a melting curve analysis (65 °C to 95 °C, 0.5 °C increment per 5 s) was performed to confirm single amplicon production. Amplification of inflammatory response markers, tight junction components, and the β-actin reference gene was conducted through real-time qPCR employing MagicSYBR Mixture (Beijing Cowin Biotech, Beijing, China). The relative expression of the target gene was calculated using the 2^−ΔΔCT^ method [25]. The values of saline-treated broilers fed the basal diet were used as a calibrator.

### 2.7. Quantitative Analysis of E. coli in the Cecal Contents

The quantification of *E. coli* levels within the cecal contents was conducted according to established protocols [26]. Briefly, the absolute quantification of populations was determined using real-time PCR in a 20 μL reaction volume: 10 μL 2 × Taq Plus Master Mix (Vazyme, Nanjing, China), 0.8 μL forward primer (5-ACTATCCCGACCGCCTTA CTG-3′), 0.8 μL reverse primer (5′-GCGCAGACCGTTTTCGCTCGG-3′), 1 μL template DNA (50 ng/μL), and 7.4 μL nuclease-free water. The amplification program was 95 °C for 5 min; 35 cycles of denaturation at 95 °C for 30 s, annealing at 58 °C for 30 s, and extension at 72 °C for 1 min; the melting curve analysis was as described above. A standard curve was generated using serial dilutions (10^8^ to 10^1^ copies/μL) of pMD18-T plasmid DNA containing the *E. coli* housekeeping gene fragment.

### 2.8. Cecum Microflora

For cecal microbial analysis, 0.2 g of cecal contents was collected from each sample and used for bacterial genomic DNA isolation using the PowerSoil DNA Isolation Kit (MO BIO Laboratories, Carlsbad, CA, USA), following the supplier’s protocol. DNA integrity and concentration were determined through spectrophotometric measurements of OD260/280 and OD260/230 ratios prior to cryopreservation at −80 °C.

The V3-V4 hypervariable regions of the 16S rRNA gene were amplified using primers 338F (5′-ACTCCTACGGGAGGCAGCAG-3′) and 806R (5′-GGACTACHVGGG TWTCTAAT-3′). PCR reactions were performed in 20 μL volumes with 10 μL 2 × Taq Plus Master Mix (Vazyme, Nanjing, China), 1 μL each primer (10 μmol/L), 2 μL DNA template (10 ng/μL), and 8 μL nuclease-free water. Amplification conditions: 95 °C for 3 min; 30 cycles of 95 °C for 30 s, 55 °C for 30 s, 72 °C for 45 s; and 72 °C for 10 min. The reaction conditions were performed as previously described [27,28]. PCR products were purified using the AxyPrep DNA Gel Extraction Kit (Axygen, Union City, CA, USA). Purified amplicons were qualified and paired-end sequenced on the Illumina MiSeq PE300 platform (Illumina, San Diego, CA, USA) according to the standard protocols of Majorbio Bio-pharm Technology Co., Ltd. (Shanghai, China).

### 2.9. Statistical Analysis

All data except for the cecum microbiota results were first tested for normality using the Shapiro–Wilk test in SPSS 19.0, and were analyzed with two-way ANOVA in SPSS 19.0, using a general linear model for a 2 × 2 factorial design. The model included *Fagopyrum dibotrys* extract, *Escherichia coli* challenge status, and their interaction as factors. Significance was defined as *p* ≤ 0.05. When significant interactions occurred, subsequent analysis employed one-way ANOVA for detailed examination.

Microbial data analysis was performed using the Majorbio Cloud [29]. The paired reads were merged into a single sequence using FLASH (v 1.2.7) software based on their overlap relationships. Concurrently, fastp (v 0.19.6) software was employed to perform quality control filtering on the reads and assess the effectiveness of the merging process. Shannon, Simpson, Chao1, and ACE indices were calculated using the boot package (version 1.3.18) in R (version 3.3.1) to evaluate microbial richness and evenness. The Kruskal–Wallis test was used to compare alpha diversity across treatment groups. Principal coordinate analysis (PCoA) based on Bray–Curtis distances was performed using the vegan package (R-3.3.1). The significance of differentiation between microbial profiles of treatments was assessed using analysis of similarity (ANOSIM). ALDEX2 (V1.30.0) was used to detect differentially abundant taxa between groups, with a false discovery rate (FDR) < 0.05 considered significant.

## 3. Results

### 3.1. Growth Performance

As illustrated in Table 3, During d 1–14 and d 15–21, dietary FDE supplementation significantly (*p* < 0.05) increased the ADG, and decreased (*p* < 0.01) the FCR. The *E. coli* challenge (d 1–14) significantly decreased (*p* < 0.05) the ADG and ADFI, and increased (*p* < 0.05) the FCR. No significant interaction (*p* > 0.05) in growth performance between the FDE and *E. coli* challenge was observed during d 1–14 and d 15–21.

### 3.2. Inflammatory Cytokines Concentrations in Serum

Serum cytokine measurements are detailed in Table 4. The *E. coli* challenge markedly (*p* < 0.05) increased serum levels of IL-1β, TNF-α, DAO, and LPS in 14-day-old broiler chickens, Conversely, dietary supplementation with FDE caused significant reductions (*p* < 0.05) in IL-1β, TNF-α, DAO, and LPS. Additionally, a notable interplay between the *E. coli* challenge and FDE supplementation was observed on IL-1β, TNF-α, DAO, and LPS parameters (*p* < 0.05). When comparing infected groups, broilers receiving FDE demonstrated substantially lower serum concentrations of IL-1β, TNF-α, DAO, and LPS relative to the COLI group.

### 3.3. Duodenum and Jejunum Morphology

As indicated in Table 5, the *E. coli* challenge caused marked decreases (*p* < 0.05) in VH/CD ratios within both duodenal and jejunal tissues while simultaneously elevating CD in the jejunum. However, adding FDE to the diet significantly increased the VH and VH/CD and reduced the CD in the duodenum. No significant interactive effects were observed between the *E. coli* challenge and FDE supplementation on the VH, CD and VH/CD of the duodenum and jejunum.

### 3.4. Intestinal Barrier-Related Gene Expression

**Figure 1:**
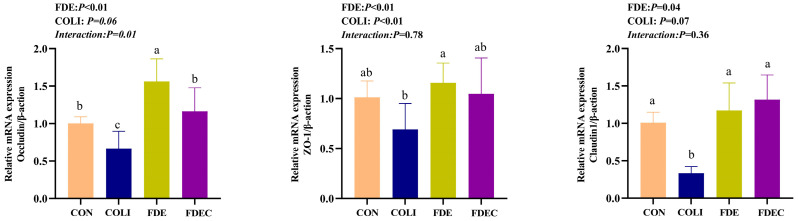
Effect of dietary *Fagopyrum dibotrys* extract supplementation on mRNA expression of tight junction proteins in the jejunum of broilers challenged with *Escherichia coli* O157. CON, fed basal diet; COLI, basal diet + *E. coli* challenge; FDE, basal diet + 500 mg/kg FDE; FDEC, basal diet + 500 mg/kg FDE + *E. coli* challenge. Different lowercase letters represent significant differences (*p* ≤ 0.05).

As shown in Figure 1, *E. coli* challenge significantly (*p* < 0.05) downregulated ZO-1 mRNA expression and showed a trend toward downregulating Occludin (*p* = 0.06) and Claudin-1 (*p* = 0.07) mRNA expression levels in the jejunum. Dietary supplementation with FDE counteracted negative effects by enhancing mRNA expression of tight junction proteins (Occludin, Claudin-1, and ZO-1) in the jejunum. A notable interaction (*p* < 0.05) was observed between COLI challenge and FDE supplementation regarding Occludin mRNA regulation, suggesting that dietary supplementation with FDE counterbalances the suppressive impact of *E. coli* challenge on Occludin gene expression in jejunum.

### 3.5. Toll-like Receptor Signaling Pathway-Related Gene Expression in the Jejunum

**Figure 2:**
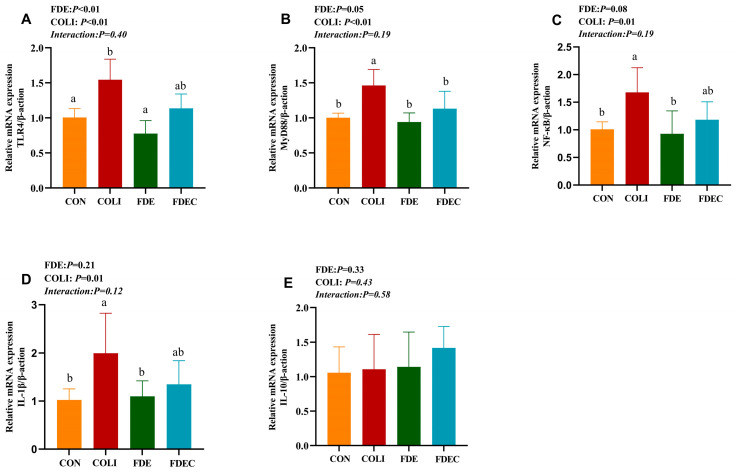
Effect of dietary *Fagopyrum dibotrys* extract supplementation on gene expressions of TLR signaling pathway-related genes in the jejunum of broiler chickens challenged with *E. coli* O157 (**A**–**E**). CON, fed basal diet; COLI, basal diet + *E. coli* challenge; FDE, basal diet + 500 mg/kg FDE; FDEC, basal diet + 500 mg/kg FDE + *E. coli* challenge. Different lowercase letters represent significant differences (*p* ≤ 0.05).

As shown in Figure 2, the *E. coli* challenge induced a significant (*p* < 0.05) upregulation of mRNA expression levels for TLR4, MyD88, NF-κB, and IL-1β in the jejunum of broiler chickens. The main effect of FDE significantly reduced (*p* < 0.05) the mRNA expression levels of TLR4, MyD88, and NF/κB in the jejunum. No statistically significant interaction (*p* > 0.05) was observed between the *E. coli* challenge and FDE supplementation regarding inflammatory factors’ gene expression of the TLR4 signaling pathway in the jejunum of broiler chickens.

### 3.6. Absolute Quantification of Escherichia coli in the Cecal Contents

**Figure 3:**
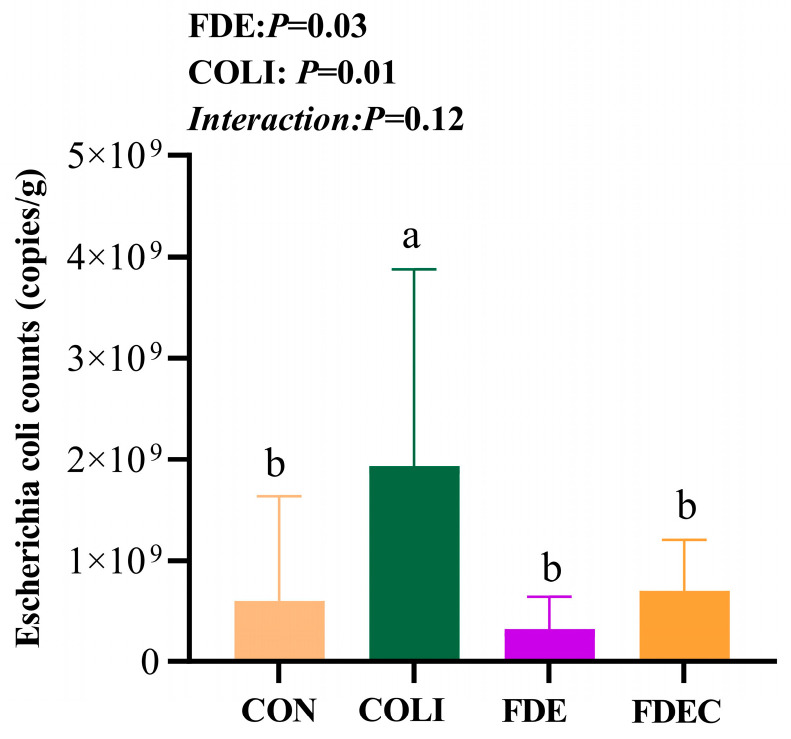
Effect of dietary *Fagopyrum dibotrys* extract supplementation on *Escherichia coli* counts in the cecal contents. CON, fed basal diet; COLI, basal diet + *E. coli* challenge; FDE, basal diet + 500 mg/kg FDE; FDEC, basal diet + 500 mg/kg FDE + *E. coli* challenge. Different lowercase letters represent significant differences (*p* ≤ 0.05).

As shown in Figure 3, the main effect of COLI challenge significantly increased the number of *E. coli* in the cecum of broiler chickens (*p* < 0.05). Dietary FDE supplementation significantly reduced the number of *E. coli* (*p* < 0.05). There was no significant interaction between *E. coli* challenge and FDE supplementation on the number of *E. coli* in the cecum (*p* > 0.05).

### 3.7. Diversity and Composition of Cecal Microbiota

**Figure 4:**
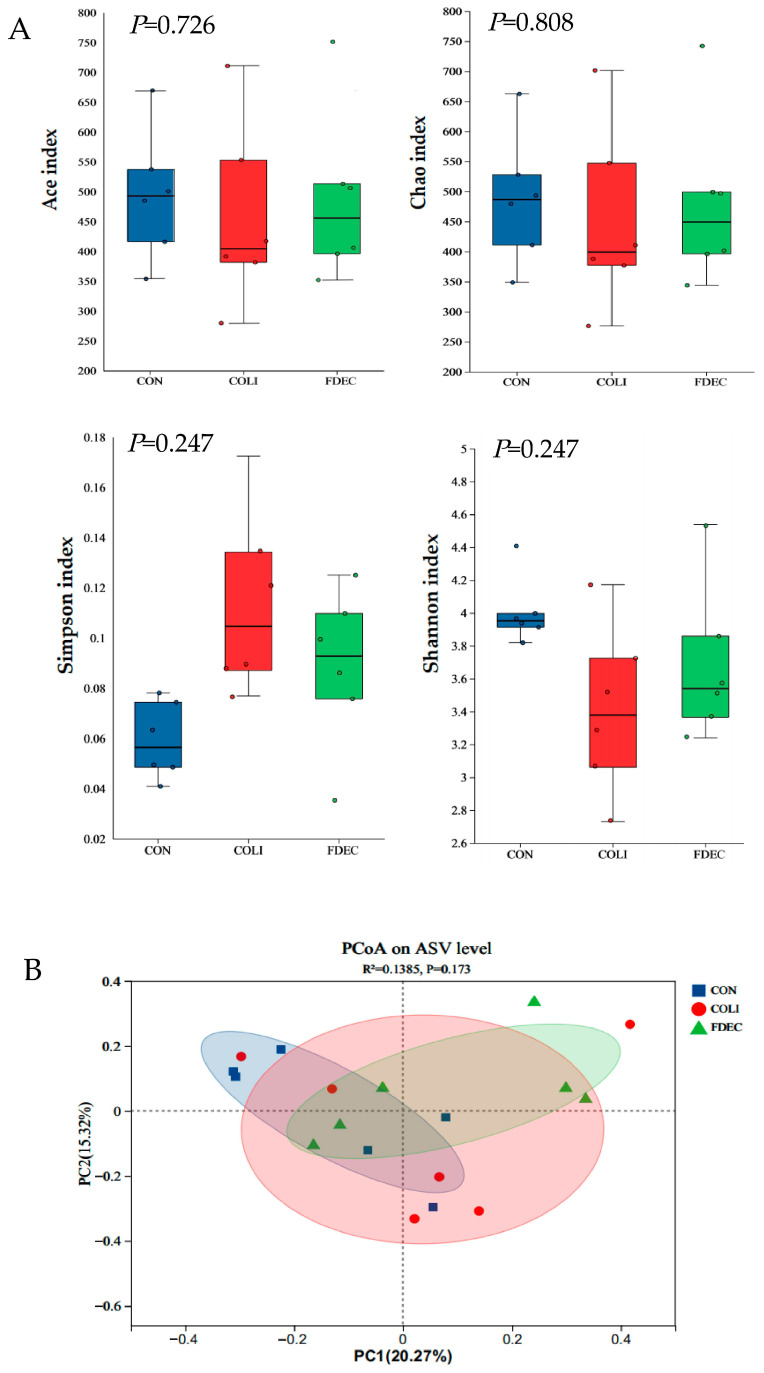
Effect of dietary *Fagopyrum dibotrys* extract supplementation on cecal bacterial diversity (α and β) in broilers (n = 6): (**A**) alpha diversity inclusive of Ace, Chao, Shannon, and Simpson indices. (**B**) beta diversity: principal coordinate analysis (PCoA) based on Bray–Curtis distances. CON, fed basal diet; COLI, basal diet + *E. coli* challenge; FDEC, basal diet + 500 mg/kg FDE + *E. coli* challenge.

The alpha diversity of the cecal microbiota was assessed using the Ace, Chao, Shannon, and Simpson indices (Figure 4A). There were no significant differences in α diversity of caecal microbiota between treatment groups (*p* > 0.05). The β-diversity PCoA analysis based on the Bray–Curtis distance revealed (Figure 4B) that sample points from each group were intermingled without distinct clustering. Further PERMANOVA testing indicated no significant differences in cecal microbiota community structure between groups (Bray–Curtis distance: R^2^ = 0.138, *p* = 0.173).

**Figure 5:**
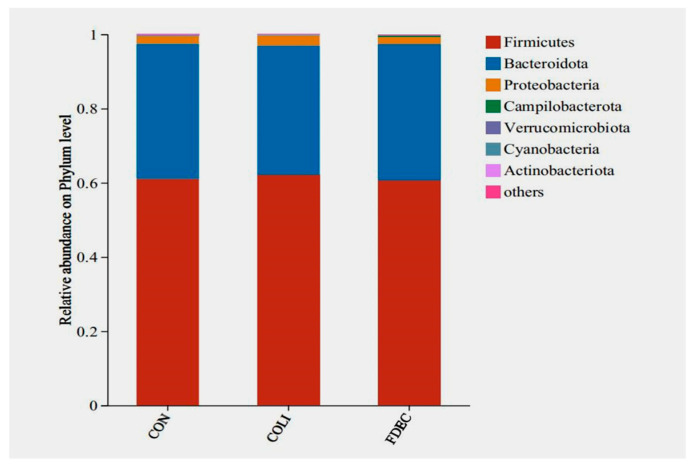
Composition of caecal microbiota of the broilers among the 3 groups at phylum level. CON, fed basal diet; COLI, basal diet + *E. coli* challenge; FDEC, basal diet + 500 mg/kg FDE + *E. coli* challenge.

**Figure 6:**
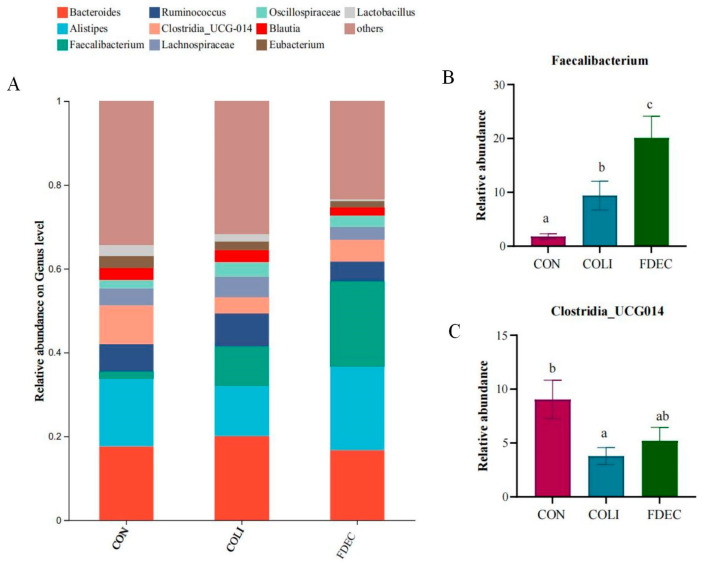
Genus-level relative abundance of microbiota from the cecal digesta of broilers: (**A**) classification of cecum flora compositions among the 3 groups at phylum level; (**B**) comparison of Firmicutes abundance among groups; (**C**) comparison of Clostridia abundance among groups. Lowercase letters in the bar graphs indicate significant differences (*p* < 0.05). CON, fed basal diet; COLI, basal diet + *E. coli* challenge; FDEC, basal diet + 500 mg/kg FDE + *E. coli* challenge.

At the phylum level, *Firmicutes* and *Bacteroidota* emerged as the predominant bacterial phyla within the cecal microbiota across all experimental groups. The relative abundance of the *Firmicutes* phylum ranged from 60.59% to 62.15% across all treatment groups, while the relative abundance of the *Bacteroidota* phylum ranged from 34.79% to 36.68%, with no statistically significant variations detected between groups (Figure 5). At the genus level, *Bacteroidetes* had the highest relative abundance in all groups, ranging from 16.67% to 20.08%. Both the *E. coli* challenge and FDE supplementation significantly increased the relative abundance of *Faecalibacterium* (Figure 6). Additionally, *E. coli* challenge induced a substantial reduction in *Clostridia_UCG-014* proportions (*p* < 0.05). Dietary supplementation with FDE demonstrated effectiveness in counteracting the pronounced alterations in *Clostridia_UCG-014* abundance triggered by the *E. coli* challenge.

## 4. Discussion

*Fagopyrum dibotrys* is a herbaceous plant with both medicinal and forage uses and is rich in bioactive compounds that contribute to animal growth and development. In this study, broiler chickens exhibited reduced appetite and impaired activity after challenge with *E. coli*. brown diarrhea was observed in the feces, and both ADFI and ADG were significantly reduced. One week later, the *E. coli* challenge also had a tendency to decrease ADG, or increase FCR. Similar studies have also reported that the *E. coli* challenge led to a decline in broiler chicken production performance [30,31,32]. These data indicated that the modeling of the *E. coli* challenge was successful. The addition of FDE to feed significantly alleviates the decline in ADG and FCR caused by *E. coli* infection. This effect may be attributed to FDE’s nutrient-rich compounds, including flavonoids with antibacterial properties, which inhibit harmful gut microorganisms, thereby enhancing nutrient absorption and boosting production performance. Similar studies have also found that FDE improves intestinal health, enhances nutrient absorption efficiency, and promotes animal growth [19].

Avian *E. coli* disease is one of the most common and harmful bacterial diseases in the poultry industry. The *E. coli* infection can lead to a series of inflammatory responses. *E. coli* entering the host’s tissues will trigger an acute inflammatory response, leading to infection and tissue damage, and the animal’s body will exhibit increased levels of acute phase proteins, inflammatory cytokines, and other substances [33,34,35,36]. This study found that *E. coli* challenge resulted in markedly elevated serum concentrations of CRP, DAO, IL-1β, and TNF-α, thereby validating the efficacy of the established experimental model. DAO, an enzyme predominantly located within the small intestinal epithelial cells, exists in substantial quantities under normal physiological conditions. Damage to intestinal villi triggers the release of this enzyme into the circulatory system, making it a reliable indicator of mucosal damage in the intestinal tract [37,38]. In this study, dietary supplementation with FDE attenuated the elevation of DAO levels induced by *E. coli* infection, which notably increased intestinal DAO activity. The intestine is one of the primary sites of colonization for Gram-negative bacteria, which were abundant in the gut. The disintegration of these microbial cells through lysis or death results in the liberation of lipopolysaccharides (LPS) into the intestinal lumen. Under normal conditions, the intestinal barrier effectively prevents LPS from entering the bloodstream. However, compromised intestinal barrier integrity facilitates LPS translocation across the intestinal epithelium into the bloodstream, subsequently elevating circulating LPS concentrations [39]. In this study, FDE supplementation reduced serum DAO and LPS levels, alongside pro-inflammatory cytokines such as TNF-α and IL-1β. These findings indicate that FDE may have direct antibacterial and anti-inflammatory effects, possibly due to the presence of its active components in the gastrointestinal tract or its various hydrolyzed products. Research in pharmacology has further confirmed the therapeutic properties of *Fagopyrum dibotrys*, including its capacity to reduce inflammation, oxidative stress, and microbial activity [11,40]. Similar studies have also reported that FDE can downregulate the expression of inflammatory factors such as TNF-α, IL-6, and IL-1β, thereby preventing and alleviating intestinal inflammation symptoms and damage in mice [41]. Thus, FDE may have a regulatory effect on intestinal permeability and intestinal damage in broiler chickens.

The impact of intestinal barrier function and mucosal injury in broiler chickens has been extensively studied. Research indicates that compromised gut integrity in commercial chickens correlates with increased pathogen translocation and systemic inflammation [42,43]. Intestinal villus height, crypt depth, and the villus height-to-crypt depth ratio were critical biomarkers for evaluating intestinal structural integrity. Both endogenous and exogenous pathogens, including bacterial and viral agents, trigger defensive responses through the intestinal barrier mechanism, safeguarding the organism from biological threats [24,44]. This study found that *E. coli* infection damaged intestinal health, specifically manifested by reducing VH and the VH/CD ratio in the jejunum of broiler chickens, with villus height decreasing, which is consistent with the results reported in previous studies.

Tight junctions between intestinal epithelial cells form a selectively permeable barrier that not only allows the transfer of nutrients, ions, and solutes but also helps maintain the integrity of the intestinal mucosal barrier and intestinal health by preventing the transport of intestinal microorganisms, antigens, and toxins into tissues. [45,46]. This research specifically examines alterations occurring in the molecular components responsible for maintaining these junctional complexes. The results showed that *E. coli* infection impaired intestinal barrier function, manifested by downregulation of Occludin and Claudin-1 mRNA expression in the jejunum. FDE supplementation upregulated the mRNA expression levels of tight junction-related genes (Occludin, Claudin-1, and ZO-1), which is associated with enhanced intestinal barrier integrity. Chen et al. (2023) further confirmed that FDE supplementation enhances tight junction protein synthesis and preserves intestinal epithelial structural stability during lipid peroxidation challenges [19]. Based on the results of serum markers, intestinal morphology and relevant gene expression, we believed that FDE supplementation protected against *E. coli*-triggered intestinal barrier damage.

This study found that FDE supplementation suppressed transcriptional activation of TLR4, MyD88, and NF-κB, consequently attenuating LPS-induced inflammatory reactions. The above results indicate that the anti-inflammatory effect of FDE on broiler chickens may be achieved by inhibiting the TLR4/NF-κB signaling pathway, thereby reducing the production of pro-inflammatory cytokines. Many studies have reported on the anti-inflammatory effects of FDE. Research has found that FDE (ethanol extract) can downregulate inflammatory responses, and alleviate LPS-induced pathological damage and tissue edema in mouse lung tissue, as evidenced by decreased pro-inflammatory factors (IL-1β, TNF-α, and IL-6) in mice. Additionally, FDE can also downregulate the expression of the TLR4 signaling pathway [15].

Research has indicated that FDE modulates cecal microbiota composition by stimulating beneficial bacterial development and restricting pathogenic bacterial expansion [47]. In a high-fat diet-fed mice model, FDE supplementation induced significant modifications in the intestinal microbial community, and promoted the proliferation of beneficial microbial species including *Lactobacillus* and *Bifidobacterium* [48]. In chick oxidative stress model, *Fagopyrum dibotrys* supplementation had a significant difference in the alpha diversity of ACE index and beta diversity. However, the results of this study demonstrated that alpha diversity (Ace, Chao, Shannon, and Simpson indices) and beta diversity (PCoA, PERMANOVA) exhibited no significant difference. This indicates that FDE exerted differing effects on the regulation of gut microbiota composition across distinct animal species or animals in varying physiological states. Although alpha diversity and beta diversity showed no significant global differences, targeted analysis revealed genus-specific changes (increased *Faecalibacterium*, alleviated *Clostridia_UCG-014* reduction). This indicates that FDE’s effect on intestinal microbiota is genus-specific rather than a global shift in community structure, which is a common regulatory pattern of plant extracts targeting key functional genera without disrupting the overall microbial balance.

## 5. Conclusions

The findings of this investigation indicated that dietary supplementation with 500 mg/kg FDE improved feed conversion efficiency, suppressed both mucosal and systemic inflammatory responses, and enhanced intestinal morphology along with Occludin and Claudin-1 gene expression of the infected broilers compared with the *E. coli*-challenged group. FDE could be used as a green feed additive to mitigate *E. coli* O157-induced gut damage and inflammation in the broiler industry. Future research should focus on determining the optimal dosage range for FDE and elucidating its in vitro antibacterial mechanism against *E. coli*, thereby providing a more comprehensive basis for the application of FDE.

## Figures and Tables

**Table 1:** Composition and nutrient levels of basal diets (as-fed basis).

Items	Ingredient (%)
Corn	53.38
Soybean meal (43%)	33.87
Soybean oil	4.30
Rapeseed meal	3.85
L-Lys·HCl (79%)	0.21
DL-Met (99%)	0.27
L-Threonine (98.5%)	0.10
Limestone	1.34
CaHPO_4_	1.69
Premix ^1^	1.00
Total	100
Nutrient levels ^2^, %	
Metabolizable energy (Mcal/kg)	3.0
CP	20.50
Lys	1.25
Met	0.58
Met + cys	0.92
Thr	0.80
Trp	0.24
Arg	1.33
Ile	0.77
Val	1.01
Ca	0.92
Available *p*	0.80
Total *p*	1.00

^1^ The premix provided the following per kg of diets: Cu 20 mg, Fe 320 mg, Mn 100 mg, Zn 200 mg, I 0.60 mg, Se 0.30 mg, Vitamin A 20,000 IU, Vitamin D 2500 IU, Vitamin E 25 mg, Vitamin K3 3 mg, Vitamin Bl 5 mg, Vitamin B2 10 mg, Vitamin B6 12 mg, Vitamin Bl2 0.03 mg, D-Pantothenic acid 20 mg, niacin 30 mg, biotin 0.10 mg, folic acid 3 mg. ^2^ The CP, Ca and TP were measured values, and metabolizable energy, effective phosphorus, lysine and methionine were calculated values.

**Table 2:** Specific sequences primers were used for qRT-PCR in this study.

Gene Target	Primer Sequence (5′ to 3′)	Accession No.	Amplicon Size (bp)
*ACTB*	F: GAGAAATTGTGCGTGACATCA	NM_205518.1	152
	R: CCTGAACCTCTCATTGCCA		
*M* *Y* *D88*	F:CCTGGCTGTGCCTTCGGA	NM_001030962	198
	R:TCACCAAGTGCTGGATGCTA		
*iNOS*	F: GCCACTTCTGAAACCCAGGTA	NM_204961.1	116
	R: ATGGCCCTTGTCCATCTCTTG		
*TLR4*	F: AGGCACCTGAGCTTTTCCTC	NM_001030693.1	96
	R: TACCAACGTGAGGTTGAGCC		
*IL* *-* *1β*	F: ACTGGGCATCAAGGGCTA	XM_015297469.2	131
	R: GGTAGAAGATGAAGCGGGTC		
*IL* *-* *10*	F:CAGACCAGCACCAGTCATCA	NM_012854.2	96
	R:TCCCGTTCTCATCCATCTTCTC		
*NF* *-* *κB*	F: GTGTGAAGAAACGGGAACTG	NM_001396038.1	203
	R: GGCACGGTTGTCATAGATGG		
*CLDN1*	F:GGTGAAGAAGATGCGGATGG	NM_001013611	139
	R:TCTGGTGTTAACGGGTGTGA		
*OCLN*	F:GATGGACAGCATCAACGACC	NM_205128	142
	R:CTTGCTTTGGTAGTCTGGGC		
*ZO-1*	F:GCCAACTGATGCTGAACCAA	XM_015278975	141
	R:GGGAGAGACAGGACAGGACT		

Abbreviations: *ACTB*, beta-actin; *MYD88*, Myeloid Differentiation Primary Response Protein 88; *iNOS*, inducible nitric oxide synthase; *TLR4*, Toll-like receptor 4; *IL-1β*, interleukins *1 beta*; *IL-10*, interleukins 10; *NF-κB*, nuclear factor-kappa B; *CLDN1*, Claudin 1; *OCLN*, Occludin1; *ZO-1*, zonula occludens protein-1.

**Table 3:** Effects of dietary *Fagopyrum dibotrys* extract supplementation on growth performance of broilers infected with *Escherichia coli* O157.

Items	Experimental Groups					Main Effect				*p*-Value		
	CON	COLI	FDE	FDEC	SEM	0	500	Non-Challenge	Challenge	FDE	COLI	Interaction
1–14 d												
BW	511.98	510.75	535.95	518.92	3.26	511.37 ^b^	528.21 ^a^	526.36	514.84	0.01	0.09	0.16
ADG (g/d)	33.64	31.85	35.28	33.97	0.01	33.29 ^b^	34.68 ^a^	34.53 ^a^	33.47 ^b^	0.03	0.02	0.52
ADFI (g/d)	41.64	39.91	42.40	41.25	0.32	40.86	41.88	42.02 ^a^	40.58 ^b^	0.07	0.02	0.60
FCR (g/g)	1.23	1.28	1.20	1.21	0.28	1.25 ^a^	1.21 ^b^	1.22 ^b^	1.24 ^a^	0.03	0.04	0.22
15–21 d												
ADG (g/d)	66.57	60.96	72.18	67.42	1.55	63.78 ^b^	70.06 ^a^	69.69	64.18	0.04	0.06	0.87
ADFI (g/d)	92.67	91.29	93.49	90.94	1.1	91.98	93.25	93.12	91.12	0.42	0.93	0.88
FCR	1.40	1.50	1.30	1.35	0.025	1.45 ^a^	1.32 ^b^	1.34	1.42	0.005	0.07	0.81

Different lowercase letters represent significant differences (*p* ≤ 0.05), and data are the mean of six replicates with 10 chickens each. Abbreviations: BW, body weight; ADG, average daily gain; ADFI, average daily feed intake; *FCR*, feed conversion ratio.

**Table 4:** Effect of dietary *Fagopyrum dibotrys* extract supplementation on serum cytokine concentration and intestinal permeability biomarkers of broilers challenged with *Escherichia coli* O157.

Items	Experimental Groups					Main Effect				*p*-Value		
	CON	COLI	FDE	FDEC	SEM	0	500	Non-Challenge	Challenge	FDE	COLI	Interaction
IL-1β (pg/mL)	320.42 ^b^	427.18 ^a^	325.82 ^b^	337.38 ^b^	11.48	380.10 ^a^	331.26 ^b^	323.66 ^b^	387.27 ^a^	0.023	0.002	0.013
TNF-α (pg/mL)	36.83 ^b^	57.52 ^a^	38.36 ^b^	41.81 ^b^	2.18	47.18 ^a^	40.43 ^b^	37.27 ^b^	51.63 ^a^	0.032	0.001	0.01
IL-10 (pg/mL)	48.09	39.95	44.25	48.29	1.36	44.21	46.38	46.36	44.12	0.392	0.435	0.075
DAO (ng/mL)	10.42 ^b^	16.08 ^a^	12.55 ^b^	11.09 ^b^	0.46	13.11 ^a^	11.82 ^b^	11.25 ^b^	14.03 ^a^	0.009	<0.01	<0.01
Endotoxin (EU/L)	31.45 ^b^	53.8 ^a^	36.27 ^b^	31.87 ^b^	1.76	42.09 ^a^	37.32 ^b^	36.27 ^b^	44.05 ^a^	<0.01	<0.01	<0.01

Different lowercase letters represent significant differences (*p* ≤ 0.05), and data are the mean of six replicates with 10 chickens each. Abbreviations: IL-1β, interleukin-1β; TNF-α, tumor necrosis factor-α; IL-10, interleukin-10; DAO, diamine oxidase.

**Table 5:** Effect of dietary *Fagopyrum dibotrys* extract supplementation on duodenum and jejunum morphology of broilers challenged with *Escherichia coli* O157.

	Experimental Groups					Main Effect				*p*-Value		
Items	CON	COLI	FDE	FDEC	SEM	0	500	Non-Challenge	Challenge	FDE	COLI	Interaction
Duodenum												
VH, μm	1923.81	1562.36	2196.86	1913.78	76.12	1706.94 ^b^	2039.59 ^a^	2060.34 ^a^	1722.10 ^b^	0.02	0.02	0.75
CD, μm	212.74	231.66	205.11	206.47	7.63	222.20	205.79	209.27	220.21	0.31	0.53	0.58
VH/CD	9.04	6.74	10.57	9.27	0.47	7.87 ^b^	10.58 ^a^	10.06 ^a^	8.62 ^b^	0.00	0.01	0.53
Jejunum												
VH, μm	1574.98	1364.99	1713.52	1414.80	46.22	1458.32	1547.57	1644.25 ^a^	1389.90 ^b^	0.21	0.00	0.55
CD, μm	205.30	258.35	192.76	215.17	9.18	234.77	205.21	199.03 ^b^	236.76 ^a^	0.09	0.03	0.33
VH/CD	7.67	5.54	8.88	6.57	0.38	6.75 ^b^	8.09 ^a^	8.74 ^a^	6.36 ^b^	0.01	0.00	0.41

Different lowercase letters represent significant differences (*p* ≤ 0.05), and data are the mean of six replicates with 10 chickens each. Abbreviations: VH, villus height; CD, crypt depth; VH/CD, the ratio of villus height to crypt depth.

## Data Availability

The data presented in this study are available on request from the corresponding author.

## References

[1] Kathayat D., Lokesh D., Ranjit S., Rajashekara G. (2021). Avian Pathogenic *Escherichia coli* (Apec): An Overview of Virulence and Pathogenesis Factors, Zoonotic Potential, and Control Strategies. Pathogens.

[2] Nawaz S., Wang Z., Zhang Y., Jia Y., Jiang W., Chen Z., Yin H., Huang C., Han X. (2024). Avian Pathogenic *Escherichia coli* (Apec): Current Insights and Future Challenges. Poult. Sci..

[3] Ghunaim H., Abu-Madi M.A., Kariyawasam S. (2014). Advances in Vaccination against Avian Pathogenic *Escherichia coli* Respiratory Disease: Potentials and Limitations. Vet. Microbiol..

[4] Joseph J., Zhang L., Adhikari P., Evans J.D., Ramachandran R. (2023). Avian Pathogenic *Escherichia coli* (Apec) in Broiler Breeders: An Overview. Pathogens.

[5] Zhang Y., Meng J., Zhang L., Bao J., Shi W., Li Q., Wang X. (2022). Shudi Erzi San Relieves Ovary Aging in Laying Hens. Poult. Sci..

[6] Wang J., Deng L., Chen M., Che Y., Li L., Zhu L., Chen G., Feng T. (2024). Phytogenic Feed Additives as Natural Antibiotic Alternatives in Animal Health and Production: A Review of the Literature of the Last Decade. Anim. Nutr..

[7] Tang Q., Chen T., Chen X., Wang C., Kuang L., Hao Z. (2025). Supplementation with Xinjiang Licorice Extract in Diets Enhanced the Growth Performance and Intestinal Immunity of Broilers. Anim. Nutr..

[8] Shanmugam S., Park J.H., Cho S., Kim I.H. (2022). Silymarin Seed Extract Supplementation En-hances the Growth Performance, Meat Quality, and Nutrients Digestibility, and Reduces Gas Emission in Broilers. Anim. Biosci..

[9] Parham S., Kharazi A.Z., Bakhsheshi-Rad H.R., Nur H., Ismail A.F., Sharif S., RamaKrishna S., Berto F. (2020). Antioxidant, Antimicrobial and Antiviral Properties of Herbal Materials. Antioxidants.

[10] Ogunkunle F., Alade O., Ogunkunle N. (2024). Pslbi-8 Effect of Sunflower (Tithonic Diversifolia) Extract on Growth Performance and Serum Antioxidant Enzymes of Broiler Chickens. J. Anim. Sci..

[11] Zhang L.-L., He Y., Sheng F., Hu Y.-F., Song Y., Li W., Chen J., Zhang J., Zou L. (2021). Towards a Better Understanding of Fagopyrum dibotrys: A Systematic Review. Chin. Med..

[12] Hu T., Zhang S., Li K., Guo Y. (2024). Selenium Nanoparticles Regulate Antioxidant Enzymes and Flavonoid Compounds in Fagopyrum dibotrys. Plants.

[13] Jing R., Li H.-Q., Hu C.-L., Jiang Y.-P., Qin L.-P., Zheng C.-J. (2016). Phytochemical and Pharmacological Profiles of Three Fagopyrum Buckwheats. Int. J. Mol. Sci..

[14] Yang J.-Y., Chen S.-Y., Wu Y.-H., Liao Y.-L., Yen G.-C. (2023). Ameliorative Effect of Buckwheat Polysaccharides on Colitis Via Regulation of the Gut Microbiota. Int. J. Biol. Macromol..

[15] Hu Y., Liu X., Song Y., Zhang Y., Li W., Zhang L., Wang A., Su Q., Yang Z., Zou L. (2024). Exploring the Anti-Inflammatory Ingredients and Potential of Golden Buckwheat (*Fagopyrum dibotrys*) on the Tlr4/Nlrp3 Pathway in Acute Lung Injury. Food Sci. Nutr..

[16] Jha R., Zhang K., He Y., Mendler-Drienyovszki N., Magyar-Tábori K., Quinet M., Germ M., Kreft I., Meglič V., Ikeda K. (2024). Global Nutritional Challenges and Opportunities: Buckwheat, a Potential Bridge between Nutrient Deficiency and Food Security. Trends Food Sci. Technol..

[17] Xiong P., Ai G., Chen J., Song W., Su W., Yu D., Song Q., Xu C., Zou Z., Wei Q. (2025). Effects of *Fagopyrum dibotrys* Rhizoma Meal Supplementation on Productive Performance, Egg Quality, Egg Nutritional Value, and Serum Biochemical Parameters of Shanma Laying Ducks. Front. Vet. Sci..

[18] Liu L., Cai X., Yan J., Luo Y., Shao M., Lu Y., Sun Z., Cao P. (2012). In Vivo and In Vitro Antinociceptive Effect of *Fagopyrum cymosum* (Trev.) Meisn Extracts: A Possible Action by Recovering Intestinal Barrier Dysfunction. Evid. Based Complement. Altern. Med..

[19] Chen Z., Dai G., Wu X., Li L., Tian Y., Tan L. (2023). Protective Effects of Fagopyrum dibotrys on Oxidized Oil-Induced Oxidative Stress, Intestinal Barrier Impairment, and Altered Cecal Microbiota in Broiler Chickens. Poult. Sci..

[20] (2004). Feeding Standard of Chicken.

[21] Mirsalami S.M., Mirsalami M. (2024). Effects of Duo-Strain Probiotics on Growth, Digestion, and Gut Health in Broiler Chickens. Vet. Anim. Sci..

[22] Kim E., Choct M., Fickler A., Pasquali G.A.M., Hall L., Crowley T.M., Sharma N.K. (2025). Supplementation of Β-Mannanase Alone or in Combination with Xylanase and Β-Glucanase Enhanced Growth Perfor-mance, Non-Starch Polysaccharide Degradation, and Gastrointestinal Environment of Broilers Offered Wheat-Based Diets. Anim. Nutr..

[23] Wu Z., Yang K., Zhang A., Chang W., Zheng A., Chen Z., Cai H., Liu G. (2021). Effects of Lactobacillus Acidophilus on the Growth Performance, Immune Response, and Intestinal Barrier Function of Broiler Chickens Challenged with *Escherichia coli* O157. Poult. Sci..

[24] Pham V.H., Abbas W., Huang J., Guo F., Zhang K., Kong L., Zhen W., Guo Y., Wang Z. (2023). Dietary Coated Essential Oil and Organic Acid Mixture Supplementation Improves Health of Broilers Infected with Avian Pathogenic *Escherichia coli*. Anim. Nutr..

[25] Livak K.J., Schmittgen T.D. (2001). Analysis of Relative Gene Expression Data Using Real-Time Quantitative Pcr and the 2−Δδct Method. Methods.

[26] Kalendar R., Hoorzook K.B., Barnard T.G. (2021). Absolute Quantification of *E. Coli* Virulence and Housekeeping Genes to Determine Pathogen Loads in Enumerated Environmental Samples. PLoS ONE.

[27] Wang Q., Cai R., Huang A., Wang X., Qu W., Shi L., Li C., Yan H. (2018). Comparison of Oropharyngeal Microbiota in Healthy Piglets and Piglets with Respiratory Disease. Front. Microbiol..

[28] Liang H., Cai R., Li C., Glendon O.H.M., Chengcheng H., Yan H. (2022). High-Throughput Sequencing of 16s Rrna Gene Analysis Reveals Novel Taxonomic Diversity among Vaginal Microbiota in Healthy and Affected Sows with Endometritis. Res. Vet. Sci..

[29] Ren Y., Yu G., Shi C., Liu L., Guo Q., Han C., Zhang D., Zhang L., Liu B., Gao H. (2022). Majorbio Cloud: A One-Stop, Comprehensive Bioinformatic Platform for Multiomics Analyses. iMeta.

[30] Lebda M.A., Mansour A.A., Elieba E.M., Hassoubah S.A., AlMalki F., El-Magd M.A., Othman S.I., Allam A.M., Tellez-Isaias G., Taha A.E. (2024). Leverage of Salvadora Persica and Pulicaria Undulata Extracts in *Escherichia coli*-Challenged Broiler Chickens. Poult. Sci..

[31] Zha P., Liu X., Zhang B., Chen Y., Zhou Y. (2025). Zinc-Loaded Aluminosilicate Minerals Improve Growth Performance and Alleviate Inflammatory Response in Broiler Chickens Challenged with Avian Pathogenic *Escherichia coli*. Poult. Sci..

[32] Meijer M.M.Y., van den Brand H., Navarro M., Roura E. (2025). The Inflammatory Response to *Escherichia coli* Lipopolysaccharide Is Mitigated by in Ovo Delivery of Carvacrol in Broiler Chicks. Poult. Sci..

[33] Watts A., Wigley P. (2024). Avian Pathogenic *Escherichia coli*: An Overview of Infection Biology, Antimicrobial Resistance and Vaccination. Antibiotics.

[34] Wei L., Liu X., Tan Z., Zhang B., Wen C., Tang Z., Zhou Y., Zhang H., Chen Y. (2025). Chlorogenic Acid Mitigates Avian Pathogenic *Escherichia coli*-Induced Intestinal Barrier Damage in Broiler Chickens Via Anti-Inflammatory and Antioxidant Effects. Poult. Sci..

[35] Yu L., Wang H., Zhang X., Xue T. (2024). Oxidative Stress Response in Avian Pathogenic *Escherichia coli*. Res. Vet. Sci..

[36] Zheng A., Bryden W., Chen X., Chen Z., Yang P., Meng K., Liu G. (2025). Lipopolysaccharide-Induced Intestinal Mucosal Injury Disrupts Proteostasis in Broiler Chickens. Ital. J. Anim. Sci..

[37] Venkitanarayanan K., Ding S., Wang Y., Yan W., Li A., Jiang H., Fang J. (2019). Effects of Lactobacillus Plantarum 15-1 and Fructooligosaccharides on the Response of Broilers to Pathogenic *Escherichia coli* O78 Challenge. PLoS ONE.

[38] Liu Y., Wang Q., Liu H., Niu J.i., Jiao N., Huang L., Jiang S., Guan Q., Yang W., Li Y. (2022). Effects of Dietary Bopu Powder Supplementation on Intestinal Development and Microbiota in Broiler Chickens. Front. Microbiol..

[39] Mohammad S., Thiemermann C. (2021). Role of Metabolic Endotoxemia in Systemic Inflammation and Potential. Front. Immunol..

[40] Zhang M., Zhang X., Pei J., Guo B., Zhang G., Li M., Huang L. (2023). Identification of Phytochemical Compounds of *Fagopyrum dibotrys* and Their Targets by Metabolomics, Network Pharmacology and Molecular Docking Studies. Heliyon.

[41] Zhu J.-h., Wang L., Xu H.-t., Ma Z.-x., Tao J.-h. (2024). Fagopyrum Cymosum Alleviates Dss-Induced Colitis Via Ameliorating Intestinal Barrier Function and Remolding Gut Microbiota. J. Funct. Foods.

[42] Zhang H., Pan S., Zhang K., Michiels J., Zeng Q., Ding X., Wang J., Peng H., Bai J., Xuan Y. (2020). Impact of Dietary Manganese on Intestinal Barrier and Inflammatory Response in Broilers Challenged with Salmonella Typhimurium. Microorganisms.

[43] Shang Q. (2024). Inulin Alleviates Inflammatory Response and Gut Barrier Dysfunction Via Modulating Microbiota in Lipopolysaccharide-Challenged Broilers. Int. J. Biol. Macromol..

[44] Celi P., Cowieson A., Fru-Nji F., Steinert R., Kluenter A.-M., Verlhac V. (2017). Gastrointestinal Functionality in Animal Nutrition and Health: New Opportunities for Sustainable Animal Production. Anim. Feed Sci. Technol..

[45] Awad W., Hess C., Hess M. (2017). Enteric Pathogens and Their Toxin-Induced Disruption of the Intestinal Barrier through Alteration of Tight Junctions in Chickens. Toxins.

[46] Lee B., Moon K.M., Kim C.Y. (2018). Tight Junction in the Intestinal Epithelium: Its Association with Diseases and Regulation by Phytochemicals. J. Immunol. Res..

[47] Zhang D., Xu Y., Chen H., Wang D., Geng Z., Chen Y., Chen Y., Xiong D., Yang R., Liu X. (2022). Fagopyrum dibotrys Extract Alleviates Hepatic Steatosis and Insulin Resistance, and Alters Autophagy and Gut Microbiota Diversity in Mouse Models of High-Fat Diet-Induced Non-Alcoholic Fatty Liver Disease. Front. Nutr..

[48] Wang D., Zhang D., Zhu Z., Zhang Y., Wan Y., Chen H., Liu J., Ma L. (2024). *Fagopyrum dibotrys* Extract Improves Nonalcoholic Fatty Liver Disease Via Inhibition of Lipogenesis and Endoplasmic Reticulum Stress in High-Fat Diet-Fed Mice. BMC Res. Notes.

